# Delayed Polyneuropathy in Farm Sprayers Due to Chronic Low Dose Pesticide Exposure

**DOI:** 10.5812/ircmj.5072

**Published:** 2014-05-05

**Authors:** Reza Boostani, Ali Mellat, Reza Afshari, Siavash Derakhshan, Morteza Saeedi, Ehsan Rafeemanesh, Maryam Mellat

**Affiliations:** 1 Neurology Department, Ghaem Hospital, Mashhad University of Medical Sciences, Mashhad, IR Iran; 2 Neurology Department, Shahid Sadoughi Hospital, Yazd University of Medical Sciences, Yazd, IR Iran; 3 Medical Toxicology Research Centre, Mashhad University of Medical Sciences, Mashhad, IR Iran; 4 Neurology Department, North Khorasan University of Medical Sciences, Bojnourd, IR Iran; 5 Occupational and Environmental Health Department, Mashhad University of Medical Sciences, Mashhad, IR Iran; 6 Physical Medicine and Rehabilitation Department, Isfahan University of Medical Sciences, Isfahan, IR Iran

**Keywords:** Pesticides, Neurotoxicity Syndromes, Polyneuropathies, Electrodiagnosis, Organophosphates

## Abstract

**Background:**

The use of organophosphates (OPs) in developing countries is rising in large quantities and non-secure methods. This problem not only causes acute poisoning but also may lead to chronic diseases such as polyneuropathy. In Iran, 60% of pesticides are organophosphate compounds that may lead to delayed polyneuropathy.

**Objectives:**

The purpose of the current study was to evaluate delayed polyneuropathy in farm sprayers due to chronic low dose pesticide exposure.

**Patients and Methods:**

In our cross-sectional study, non-randomized sampling method was performed and 100 farm sprayers (cases) and 100 hospital personnel (controls) after precise systemic and neurological examination were recruited to this study from June 2011 to august 2011. The nerve conduction studies were performed and these indices were recorded: Compound Muscle Action Potential (CMAP), amplitude and Distal Latency (DL) and Nerve Conduction Velocity (NCV) of common peroneal nerve, Peak Latency (PL) and amplitude of Sensory Nerve Action Potential (SNAP) and Nerve Conduction Velocity (NCV) of sural and radial sensory nerves.

**Results:**

Among 100 cases, 55 farm sprayers complained of non-neurological problems including: ophthalmologic, dermatologic and pulmonary complications. The ophthalmologic complaints (44%) were the most. The mean peroneal CMAP amplitude and NCV, sural PL, radial SNAP amplitude, PL and NCV in the case group were significantly different compared to control group. Mean exposure time to OPs in farm sprayers without neurological problem (40%) was 11.81 ± 5.84 years but in farm sprayers with neurological problems (60%) was 15.70 ± 9.08 years, which represents the effect of OPs exposure duration on neurologic problems.

**Conclusions:**

Chronic low dose pesticide exposure could lead to delayed peripheral neuropathy as well as systemic (skin, eyes and lungs) complications. In farm sprayers electrodiagnostic indices were significantly abnormal as compared to control group. The normal indices did not rule out neurologic involvement and it seems that measurement of these indices at the beginning of the farm sprayers employment and intermittently during their work is helpful for detecting delayed polyneuropathy.

## 1. Background

Huge amounts of organophosphates pesticides (OPs) are used worldwide. Recommended agricultural pesticides, which are used in the developing countries, have more problems such as high doses consumption and often are used in non-secure applications and without appropriate protective equipments (1). Acute OPs poisoning causes a wide range of morbidity and mortality (2) and is more frequent in developing countries, where almost all deaths due to pesticide poisoning happens (3). In Iran, 4.6% of all acute poisonings are related to pesticides (4, 5) whereas in recent years, these poisonings have been gradually declined (6). In Iran, more than 60% of pesticides used in agricultural and industrial settings are OPs (7). Also in Iran, based on local reports, farms are sprayed against insects 5 times per year and the most prevalent method for plants and gardens protection is chemical, and the most common chemical agents are Ops (7). Most popular OPs used in Iran are:

Teflubenzuron, Phosalone, Phosalone, Indoxacarb, Diazinon, Azinphosmethyl, Bromopropylate and Oxydemeton-methyl (7). Acute exposure to OPs may lead to numerous toxic complications, including acute cholinergic clinical episodes (dizziness, anxiety, headache, nausea, rhinorrhoea, sweating, salivation, lachrymation, bronchorrhea, bronchospasm, pulmonary edema, abdominal pain, diarrheal, miosis, tachycardia, hypertension, muscle twitching, fasciculation, Loss of consciousness, convulsions, flaccid paralysis, deep coma and cardiopulmonary arrest), the intermediate syndrome, organophosphate-induced delayed polyneuropathy (OPIDP) and chronic neurological effects (8, 9).

OPIDP is a symmetrical sensori-motor axonopathy, which is more severe in long axons and occurs 7-14 days after exposure which could be extremely disabling and electrodiagnostic studies show sensory nerve dysfunction of the lower extremities more than motor nerves (9). Acute toxicity effects are produced by the irreversible inactivation of the enzyme cholinesterase. The intermediate syndrome’s mechanism is not understood precisely, while OPIDP is caused by the inhibition and subsequent aging (dealkylation) of neuropathy target esterase (NTE) (8, 10). Additionally, there are some evidences for the ability of OPs causing disruption in a number of metabolic and cell signaling pathways that affect neuronal cell proliferation, differentiation and survival and interaction with non-esterase proteins such as tubulin (11). However, the possible effects of long-term low-level exposure to OPs remain unclear (8). Chronic OPs poisoning is usually occupational which may occur in workers with daily exposure and is most common among farm sprayers and sheep divers (9). Majority of OPs compounds are metabolized rapidly and excreted. However, since several OPs compounds cause slowly reversible inhibition of cholinesterase, accumulation of this effect can occur (9). The farm sprayers who exposed to OPs revealed lower AChE activity and higher urinary dialkylphosphate and 8-hydrox-29-deoxyguanosine than the controls (9). Also they had higher DNA damage than the controls (12). Some complications due to chronic low dose exposure to OPs are parkinsonism (13) and psychiatric diseases (14). Also neurobehavioral changes due to chronic exposure to low doses of OPs have been reported (15-17), although these results have not been supported by other studies (12). Some studies have shown that OPs exposure could lead to neurodevelopmental adverse effect on Mexican-American children (18) and may contribute to Attention-Deficit/Hyperactivity Disorder (ADHD) prevalence in American children (19). This study was performed to determine the role of chronic low dose exposure with OPs in developing polyneuropathy in Khorasan-Razavi province farm sprayers.

## 2. Objectives

The purpose of the current study was to evaluate delayed polyneuropathy in farm sprayers due to chronic low dose pesticide exposure.

## 3. Materials and Methods

In our cross-sectional study, non-randomized sampling method was performed and all registered farm sprayers in Occupational & Environmental Department of Khorasan-Razavi Province of Iran (n = 142) were recruited to this study. Out of these cases, 118 (82%) farm sprayers agreed to participate in this study from June 2011 to August 2011. 16 cases (11%) were excluded due to underlying diseases including diabetes, thyroid disease, chronic low back pain, history of brucellosis, acquired or hereditary poly neuropathy and disk herniation. Two females (1%) were excluded for more matching between case and control groups. Also 100 healthy male employees’ in Ghaem Medical and Research Center (Hospital) of Mashhad city of Iran with no underlying disease or occupational exposure to toxins were enrolled in our study as the control group. Clinical and electrodiagnostic evaluations were done in the neurology department of Ghaem Medical and Research Center, which is a referral university hospital. Moreover for omitting measurement bias, only a faculty member did all observations and measurements rechecked precisely.

All cases and controls underwent obtaining precise history and complete systemic and neurologic examination including sensory (light touch, pinprick, temperature, proprioception and vibration sensations) and motor examinations (muscular tone and force, deep tendon reflexes).

Electrodiagnostic indices which were recorded by calibrated TOENNIES electromyography machine (Toennies Company-Germany) included: compound muscle action potential (CMAP) amplitude, distal latency (DL) and nerve conduction velocity (NCV) of common peroneal nerve, peak latency (PL), amplitude of sensory nerve action potential (SNAP) and NCV of sural and radial sensory nerves.

In peroneal motor nerve conduction study (NCS), proximal and distal stimulations were performed at fibular neck and ankle respectively. The indices were recorded from extensor digitorum brevis muscle. Sural NCS was performed by stimulation of nerve trunk at a distance 14 cm from lateral ankle border where recording electrodes were placed. For radial sensory nerve, recording electrodes were placed on the dorsum of the hand in the radial sensory nerve territory. The stimulation was carried out at 12 cm from recording electrodes in distal forearm. In all sensory NCS, the active and reference electrodes distance was about 3 to 4 cm. All stimulations were supra maximal. Normal values for the nerve conduction measures were taken from standard published norms.

The case group was also divided into two subgroups: with neurological problems (n = 40) and without neurological problems (n = 60). These two subgroups were compared separately. The data were checked for normal assumption using Kolmogorov–Smirnov test and none of variables were significant at 0.05. Finally, data were analyzed using Student t-test and Chi-square test by SPSS version 11.5 software. P values less than 0.05 were considered statistically significant. Ethics approval was obtained from Ethics Committee of the Mashhad University of Medical Sciences (No. 2182). All enrolled subjects completed informed consent.

## 4. Results

### 4.1. Demographic Features

Overall 100 farm sprayers were evaluated clinically and electrodiagnostically and compared to 100 healthy hospital personnel (controls). All subjects were males. The mean age ± standard deviation of cases was 43.4 ± 6.5 years, which was significantly higher than the control group (40.7 ± 6 years) (P = 0.002) (Table 1) (see limitation please). In cases group, age had no difference significantly between farm sprayers with and without neurologic findings (P = 0.153).

**Table 1.** Age Distribution in Cases and Controls ^a^

Groups	Age, y	P value
**Cases (n = 100)**	43.4±6.5^a^	0.002
**Controls (n = 100)**	40.7±6.0	
**Cases with neurologic problems (n = 40)**	44.6±6.3	0.153
**Cases without neurologic problems (n = 60)**	42.7±6.6	

^a^ Data are presented as Mean±SD.

### 4.2. Clinical Findings

#### 4.2.1. Systemic Findings

In 100 cases, systemic complications were: ophthalmologic (burning and itching sensation, redness and lacrimation) (44%), dermatologic (hypersensitivity, burning and itching sensations) (40%) and pulmonary (cough and dyspnea) (32%) (Table 2). It should be emphasized that because of complications overlaps, overall 55 cases had systemic complications.

**Table 2.** Prevalence of Complications in 100 Farm Sprayers ^**a**^

Complications	Frequency, %
**Neurologic**	
Sensory symptoms	37 (37)
Sensory signs	16 (16)
Motor symptoms	2 (2)
Motor signs	0 (0)
**Systemic**	
Ophthalmologic	44 (44)
Dermatologic	40 (40)
Pulmonary	32 (32)

^a^ Some overlaps exist between complications.

#### 4.2.2. Neurologic Findings

Among cases, neurologic findings were divided to: sensory symptoms (paresthesia and numbness in glove and stocking pattern) (37%), sensory signs (decreased sense of pain and temperature) (16%), motor symptoms (distal weakness of four limbs) (2%), and no motor signs (force, tone and reflexes) (0%) (Table 2). It should be emphasized that because of complications overlaps, overall 40 cases had neurologic complications.

### 4.3. Electrodiagnostic Findings

#### 4.3.1. Cases Versus Controls

##### 4.3.1.1. Mean Values of Electrodiagnostic Indices

Mean (SD) of peroneal CMAP amplitude (P = 0.013) and NCV (P < 0.001), sural PL (P = 0.006), radial SNAP amplitude (P < 0.001), PL (P < 0.001) and NCV (P = 0.046) in cases and controls were significantly different. Sural NCV and peroneal CMAP Distal latency were not different between cases and controls. All mentioned indices, however, were within normal ranges (Table 3).

**Table 3.** Comparison of Electrodiagnostic Indices Between Cases and Controls, and Frequency of Abnormal Subjects ^a^

Index	Mean ± SD			Number of Abnormal Subjects		
	Cases	Controls	P value	Cases	Controls	P value
**Peroneal**						
CMAP amplitude, mv	5.4 ± 2.5	6.4 ± 1.9	0.013	5	3	0.721
CMAP distal latency, ms	4.8 ± 1.1	5 ± 0.9	0.435	4	2	0.683
NCV, m/s	46.8 ± 5.6	52.9 ± 5.5	< 0.001	24	2	< 0.001
**Sural**						
SNAP amplitude, mv	12.5 ± 4.8	13.7 ± 4.7	0.090	6	0	0.029
SNAP peak latency, ms	3.5 ± 0.5	3.3 ± 0.5	0.006	0	0	> 0.999
NCV, m/s	55.8 ± 10.0	56.5 ± 10.1	0.669	0	0	> 0.999
**Radial**						
SNAP amplitude, mv	17.9 ± 7.4	21.9 ± 7.8	< 0.001	0	0	> 0.999
SNAP peak latency, ms	2.6 ± 0.4	2.4 ± 0.4	< 0.001	15	1	< 0.001
NCV, m/s	58.7 ± 8.7	61.1 ± 8.1	0.046	14	1	< 0.001

^a^ Abbreviations: CMAP, compound muscle action potential; NCV, nerve conduction velocity; SNAP, sensory nerve action potential.

##### 4.3.1.2. Frequency of Abnormal Electrodiagnostic Indices

Abnormal peroneal NCV (m/s) was observed in 24% of cases which was significantly higher than controls (2%) (P < 0.001). Also abnormal sural SNAP amplitude (mv) observed in 6% which was significantly higher than controls (P = 0.029). Abnormal radial SNAP peak latency (ms) and radial NCV (m/s) were also more frequent in cases (P < 0.001). Frequencies of abnormal findings of other indices were not different significantly between two groups (Table 3).

#### 4.3.2. Cases With Neurologic Problems Versus Cases Without Neurologic Problems

In total, 40% farm sprayers had neurologic problems (N+). Electrodiagnostic indices of these cases were separately compared to data obtained from farm sprayers without neurologic problems (N-) (Table 4). The mean age in farm sprayers with and without neurologic problems were 44.6 (6.4) and 42.7 (6.6) years respectively without significant difference. Duration of exposure to pesticides was significantly longer in (N+) cases compared to (N-) cases (P = 0.036) (Table 5).

**Table 4.** Duration of Organophosphates Exposure (years) in Farm sprayers ^a^

Cases With Neurologic Complications (n = 40)	Cases Without Neurologic Complications (n = 60)	P value
15.7 ± 9.1	11.9 ± 6.5	0.036

^a^ Data are presented as Mean±SD.

**Table 5.** Electrodiagnostic Indices and Frequency of Abnormal Subjects in Farms Prayers With (N+) (n = 40) and Without (N‑) (n = 60) Neurologic Complications[Table-fn fn10772][Table-fn fn10773]

Index	Mean ± SD			Abnormal Subject		
	N+	N–	P Value	N+	N–	P Value
**Peroneal**						
CMAP amplitude, mv	4.2 ± 1.5	4.8 ± 2.2	0.105	1 (2.5)	4 (6.7)	0.640
CMAP distal latency, ms	5.2 ± 0.6	4.6 ± 1.3	0.008	1 (2.5)	3 (5)	0.916
NCV, m/s	45.2 ± 3.1	47.9 ± 6.6	0.006	7 (17.5)	17 (28.3)	0.316
**Sural**						
SNAP amplitude, mv	12.1 ± 4.2	12.8 ± 5.1	0.507	3 (7.5)	3 (5)	0.933
SNAP peak latency, ms	3.6 ± 0.4	3.1 ± 0.5	< 0.001	0 (0)	0 (0)	> 0.999
NCV, m/s	50.2 ± 5.2	59.9 ± 10.7	< 0.001	0 (0)	0 (0)	> 0.999
**Radial**						
SNAP amplitude, mv	14.9 ± 4.7	19.8 ± 8.2	< 0.001	19 (47.5)	16 (26.7)	0.054
SNAP peak latency, ms	2.8 ± 0.4	2.5 ± 0.3	< 0.001	13 (27.5)	2 (3.3)	< 0.001
NCV, m/s	55.2 ± 6.7	61.1 ± 9.1	< 0.001	9 (22.5)	5 (8.3)	0.088

^a^ Abbreviations: CMAP, compound muscle action potential; NCV, nerve conduction velocity; SNAP, sensory nerve action potential.

^b^ Data are presented as Mean±SD or No. (%).

##### 4.3.2.1. Mean Values of Electrodiagnostic Indices

Mean (SD) of peroneal CMAP distal latency (P = 0.008) and NCV (P = 0.006), sural SNAP peak latency (P < 0.001) and NCV (P < 0.001), radial SNAP amplitude (P < 0.001), peak latency (P < 0.001) and NCV (P < 0.001) in cases with neurologic problems and cases without neurologic problems were significantly different. Peroneal CMAP amplitude and sural SNAP amplitude were not different among these cases. All mentioned indices, however, were within normal ranges (Table 5).

##### 4.3.2.2. Frequency of Abnormal Electrodiagnostic Indices

Frequency of abnormal indices in all beside radial SNAP peak latency (P < 0.001), were not different significantly between two groups.

## 5. Discussion

In our study, ophthalmologic, dermatologic and pulmonary complications were 44%, 40% and 32% respectively, which were rather higher than an Indian report in which 23.5% had skin complaints, 17.6% had gastrointestinal complaints and 8.8% had ophthalmologic complaints (20). These differences could be related to potential higher or longer exposure to pesticides and lack of adequate protection by our farm sprayers.

In similar studies from the US and UK, a range of 2 to 31% of exposed cases complained of intermittent sensory symptoms mainly in the form of the glove and stocking (12, 21, 22). Although no impaired sensation was found in one study (21), impaired temperature sensation thresholds have been reported in up to 65% of cases (12, 22). Similarly, in these studies a range of 0 to 21% of farm sprayers complained of motor weakness (12, 21, 22). In our study, 37% of cases complained of sensory symptoms, 16% had sensory signs and just 2% had motor symptoms, which were similar to other studies (Table 2).

In the current study, 40% of farm sprayers had neurological findings (signs and symptoms). The mean duration of exposure to pesticides was longer in the group with neurologic problems compared to farm sprayers without neurologic problems (Table 4). This could be consistent with the effect of dose-time exposure of pesticides to peripheral nerves. As far as we know, this is the first report of this kind. We also found that a quarter of cases have isolated neurological problems. This might raise the importance of regular evaluation of cases.

We found that mean values of all, except peroneal CMAP distal latency, sural SNAP amplitude and sural NCV, were longer in cases in comparison to controls. Despite this, both groups mean values were within normal ranges. As a result, any normal nerve electrodiagnostic indices could not rule out peripheral nerves damages due to chronic organophosphates exposure. So baseline values should be obtained in these cases at the beginning of the employment.

Also, frequencies of abnormal electrodiagnostic indices were higher for Radial NCV, radial SNAP peak latency, sural SNAP amplitude and peroneal NCV. This is rather similar to Jamal et al. study (22) but inconsistent with no abnormal electrodiagnostic findings in Albers et al. study (21).

Generally indices related to radial sensory nerve were more prominent in difference between cases and controls. This may be due to more exposure and less protection of upper limb during spraying. This also could suggest the radial sensory nerve as the best nerve to be investigated for baseline and follow up studies.

Based on Table 5, majority of indices obtained from farm sprayers with neurologic complications were different significantly compared to farm sprayers without neurologic complications. Among these indices, radial sensory nerve indices were more different. It means that radial sensory nerve is more susceptible to chronic pesticides exposure than other nerves.

This study reveals that exposed farm sprayers to OPs in a time related manner may lead to abnormal systemic (ophthalmologic, dermatologic and pulmonary), neurologic (e.g. glove and stocking paresthesia) and electrophysiological abnormalities (mainly radial sensory nerve’s indices). Baseline and follow up measurements of electrophysiological indices with a focus on radial sensory nerve, applying more respiratory, eye and skin protections are recommended. This study proved that the normal electrodiagnostic indices in agriculture sprayers necessarily does not mean the absence of neuropathy, so comparing indices periodically in farm sprayers can help to identify many of these sprayers with delayed neuropathy. Therefore, it is suggested that sprayers working in farms in addition to routine examination before beginning their work, basic electrodiagnostic assessment also should be considered. In this study, mean age of cases and controls were significantly different. As electrodiagnostic indices are not affected by age below 60 years this could have no impact on these findings. No NTE enzymatic level measurement was performed.
